# HSV-2- and HIV-1- permissive cell lines co-infected by HSV-2 and HIV-1 co-replicate HSV-2 and HIV-1 without production of HSV-2/HIV-1 pseudotype particles

**DOI:** 10.1186/1743-422X-4-2

**Published:** 2007-01-05

**Authors:** Jérôme LeGoff, Hicham Bouhlal, Maxime Lecerf, Christophe Klein, Hakim Hocini, Ali Si-Mohamed, Martin Muggeridge, Laurent Bélec

**Affiliations:** 1 Université Paris V, Equipe « Immunité et Biothérapie Muqueuse », Unité INSERM Internationale U743 (« Immunologie Humaine »), Centre de Recherches, Biomédicales des Cordeliers, & Laboratoire de Virologie, Hôpital Européen Georges Pompidou, Paris, France; 2 Service Commun d'Imagerie Cellulaire et de Cytométrie, INSERM IFR58, Centre de Recherches Biomédicales des Cordeliers, Paris, France; 3 Department of Microbiology and Immunology, Louisiana State, University Health Sciences Center, Shreveport, LA 71130, USA

## Abstract

**Background:**

Herpes simplex virus type 2 (HSV-2) is a major cofactor of human immunodeficiency virus type 1 (HIV-1) sexual acquisition and transmission. In the present study, we investigated whether HIV-1 and HSV-2 may interact at the cellular level by forming HIV-1 hybrid virions pseudotyped with HSV-2 envelope glycoproteins, as was previously reported for HSV type 1.

**Methods:**

We evaluated in vitro the production of HSV-2/HIV-1 pseudotypes in mononuclear CEM cells and epithelial HT29 and P4P cells. We analyzed the incorporation into the HIV-1 membrane of HSV-2 gB and gD, two major HSV-2 glycoproteins required for HSV-2 fusion with the cell membrane, in co-infected cells and in HIV-1-infected P4P cells transfected by plasmids coding for gB or gD.

**Results:**

We show that HSV-2 and HIV-1 co-replicated in dually infected cells, and gB and gD were co-localized with gp160. However, HIV-1 particles, produced in HIV-1-infected cells expressing gB or gD after transfection or HSV-2 superinfection, did not incorporate either gB or gD in the viral membrane, and did not have the capacity to infect cells normally non-permissive for HIV-1, such as epithelial cells.

**Conclusion:**

Our results do not support the hypothesis of HSV-2/HIV-1 pseudotype formation and involvement in the synergistic genital interactions between HIV-1 and HSV-2.

## Background

Genital infection by herpes simplex virus type 2 (HSV-2) is considered one of the major cofactors favoring both sexual transmission and acquisition of the human immunodeficiency virus type 1 (HIV-1) [1]. This assertion is based on several criteria as defined by Judith and Wasserheit [2], including mainly epidemiological evidence, biological plausibility, and ongoing intervention studies [1,3,4]. The biological arguments of HSV-2 as a major cofactor of HIV-1 sexual transmission include: (i) Disruption of the mucosal integrity secondary to herpetic ulcerations; (ii) In vivo association between the genital loads of HIV-1 RNA and HSV-2 DNA in female genital secretions among HSV-2 asymptomatic and symptomatic shedders [5-7], and observation of increased genital shedding of HIV reported in men with genital ulcers caused by HSV-2 [8]; (iii) In vitro evidence that HIV-1 and HSV-2 synergistically interact [9-13].

It has been recently hypothesized that HIV-1 and HSV-1 may interact at the cellular level by the formation of HIV-1 hybrid virions pseudotyped with herpes simplex virus type 1 (HSV-1) envelope glycoproteins [14,15]. Thus, two independant studies showed that cell-free culture supernatant from HIV-1 and HSV-1 dually infected lymphocyte cell lines was able to induce HIV-1 productive infection in cells normally resistant to HIV-1 infection, leading to the proposal that HIV-1 particles may acquire HSV-1 glycoproteins through budding at the cell surface [14,15]. Such pseudotyped HIV-1 virions acquired new phenotypic properties with a larger cell tropism. Especially, this would allow the infection of normally non permissive cells by fusion of the viral envelope with the cell membrane by a mechanism depending on HSV-1 proteins. Such hybrid virions could have a modified, likely increased tropism for target cells within the genital mucosa. In vivo observations obtained by electron microsopy from herpetic skin lesions showed that CD4 negative epidermal keratinocytes contained HIV-1 and HSV-1 particles, supporting the hypothesis of the presence of HIV-1 pseudotyped virions [16].

The interactions between HSV-2 and HIV-1 at the cellular level have been poorly analyzed, and the formation of pseudotyped particles between the two viruses has not been yet evaluated. The aim of the present study was to extend the previous preliminary reports on HSV-1/HIV-1 pseudotype production, to HSV-2 using a variety of epithelial and mononuclear cell lines, and to analyze the incorporation into the HIV-1 membrane of gB and gD, two major HSV-2 glycoproteins required for HSV-2 fusion with the cell membrane.

## Results

### Colocalization of HSV-2 gB and gD with gp160 in co-infected or transfected cells

**Figure 1:**
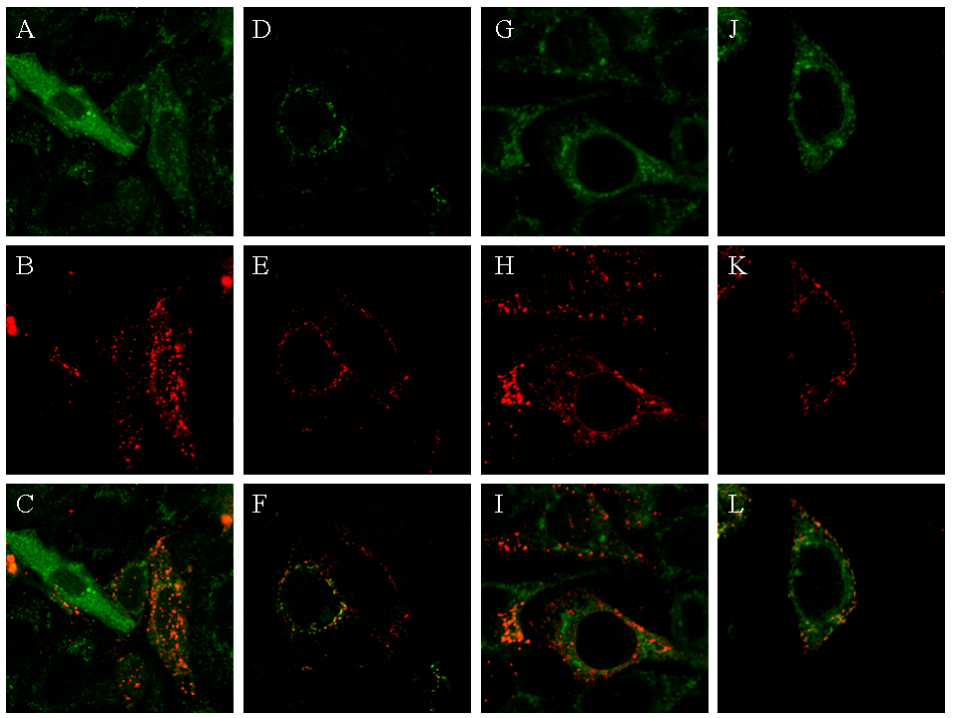
**Co-staining of HSV-2 gB or gD with HIV-1 gp160**. HIV-1 infected P4P cells were either superinfected by HSV-2 (A-F) or transfected by a plasmid coding for gB (G-I) or gD (J-L). Cells were stained by immunofluorescence for the co-detection of gp160 and gB (A-C, G-I) or gp160 and gD (D-F, J-L), using human polyclonal anti-gp160 followed by FITC-labelled F(ab')2 rabbit anti-human IgG giving a green staining for HIV-1 gp160 and mouse Mabs against gB or gD followed by a TRITC-labelled rabbit anti-mouse IgG giving a red staining for HSV-2 gB or HSV-2 gD.

Prior evaluating the potential formation of pseuotyped particles, we first analyzed at the cellular level the co-expression of gB and gp160, or gD and gp160, in HIV-1 infected P4P cells superinfected by HSV-2, and in HIV-1-infected P4P cells transfected by plasmids coding for gB or gD, by using confocal microscopy. Our observations confirm that P4P cells exposed to both HIV-1 and HSV-2 can be co-infected and coreplicate the two viruses, since we can detect in the same cell glycoproteins from both viruses. HIV-1 P4P cells transfected with plasmids coding for gB or gD also express both the HSV-2 glycoprotein and gp160 (Figure 1). In addition, as shown in Figure 1, the superposition of fluorescence signals indicates that gB and gD may co-localize with HIV-1 gp160 in both co-infected and transfected P4P cells. The pattern of staining in single infections with either HIV-1 or HSV-2 was the same as that observed in co-infected cells (not shown).

### CEM cells are susceptible to HSV-2 and HIV-1 co-infection

Prior HSV-1 infection of CEM cells has been shown to result in a delay of HIV-1 production, due to cell growth inhibition and apoptosis, while HSV-1 superinfection of HIV-infected CEM cells promoted HIV expression [17]. We have therefore focused our analysis on this latter condition.

**Figure 2:**
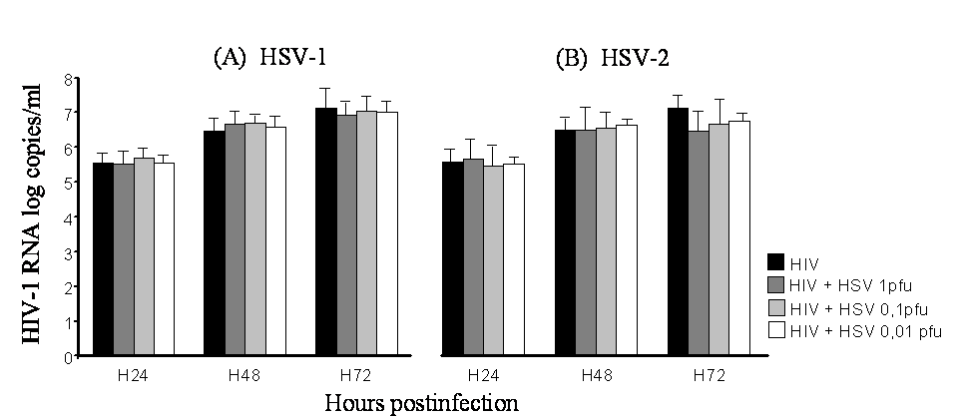
**HIV-1 replication in HIV-1 infecetd CEM cells co-infected by HSV-1 or HSV-2**. HIV-1 RNA has been quantified in the supernatant of CEM cells every 24 h up to 72 h following superinfection by HSV-1 (A) or HSV-2 (B) at an MOI of 1 (dark grey), 0.1 (light grey) and 0.01 (white). Cells not superinfected are represented in black. Values are expressed in log_10 _copies/ml. Data are representative of three independent experiments.

**Figure 3:**
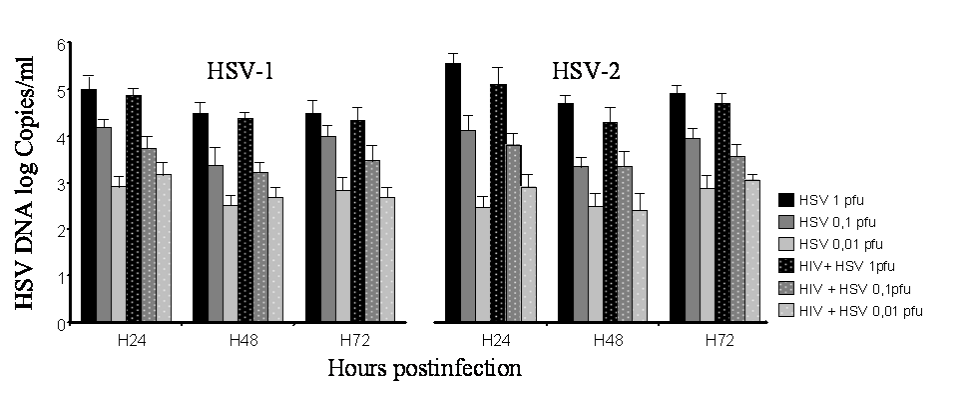
**HSV-1 and HSV-2 replication in CEM cells co-infected by HIV-1**. HSV-1 (A) and HSV-2 (B) viral load, expressed in log_10 _copies/ml, has been determined in the supernatant of CEM cells infected by HSV-1 or HSV-2 at an MOI of 1 (dark grey), 0.1 (light grey) and 0.01 (white) and in HIV positive CEM cells superinfected by HSV-1 or HSV-2 at an MOI of 1 (dotted dark grey), 0.1 (dotted light grey) and 0.01 (dotted white) for up to 72 h.

CEM cells infected by HIV-1NDK strain were superinfected by HSV-1 or HSV-2 at a multiplicity of infection (MOI) of 0.01, 0.1 or 1 pfu/cell for 72 h. Every each 24 hours, culture supernatant was collected for viral quantifications by real time PCR, and after washing cells were reincubated with fresh medium. Our results show that HSV-1 and HSV-2 did not modify the production of HIV-1 RNA, and that HIV-1 did not influence either HSV-1 or HSV-2 replication in CEM cells (Figures 2 and 3). The cell free supernatants of dually infected CEM cells were then used for the infection of Vero cells. In the first 48 h, cytopathic effect related to HSV-2 was observed by optical microscopy (not shown). No HIV-1 p24 antigen was detected in the supernatant of Vero cells at 24 h or 48 h, and DNA of the Vero cell pellet contained no HIV-1 proviral DNA as detected by real time PCR (not shown). The HSV-1 and HIV-1- co-infected CEM cells and HSV-2- and HIV-1- co-infected CEM cells did not lead to the production of HIV-1 virions able to infect cells that are usually non-permissive.

### HIV-1 particles produced from HIV-1 and HSV-2 co-infected epithelial cells do not infect Vero cells

**Figure 4:**
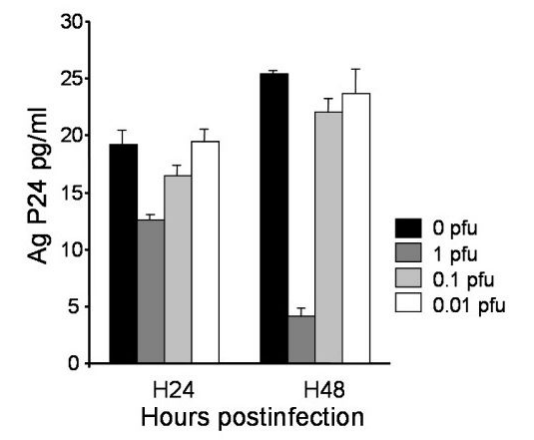
**HIV-1 expression in dually HIV-1 and HSV-2 infected HT29 cells**. HIV-1 production in infected HT29 cells 24 h and 48 h after HSV-2 superinfection at the MOI of 1 (dark grey), 0.1 (light grey) and 0.01 (white). Values, expressed in pg/ml of p24 Ag, are the mean of three independent experiments. Control HIV-1 infected HT29 cells not superinfected by HSV-2 are represented in black.

Intestinal HT29 cells are epithelial cells susceptible to infection by both R5 and X4 HIV-1 strains [18]. HT29 cells were infected with HIV-1NDK for 48 h before HSV-2 superinfection at MOIs of 0.01, 0.1 and 1. HIV-1 production was assessed at 24 and 48 h following the infection. At 24 h, the medium was removed for viral quantification and was replaced with fresh medium for the 48 hour time-point. The results depicted in Figure 4 show that HIV-1 production by HT29 cells decreased significantly following HSV-2 superinfection at a MOI of 1 pfu after 24 h (P = 0.04). The influence of HSV-2 at a MOI of 1 pfu on the decrease of HIV replication was more pronounced 24 hours later. In contrast, lower HSV-2 inoculum (0,1 and 0.01 pfu) did not seem to inhibit HIV-1 production in a 48 h delay. Supernatants of HIV-1 and HSV-2 dually infected HT29 cells collected at 24 and 48 h did not lead to HIV-1 infection of Vero cells as determined by detection of proviral DNA, while HSV-2 replication was confirmed by the detection of typical cytopathic effect (not shown).

**Figure 5:**
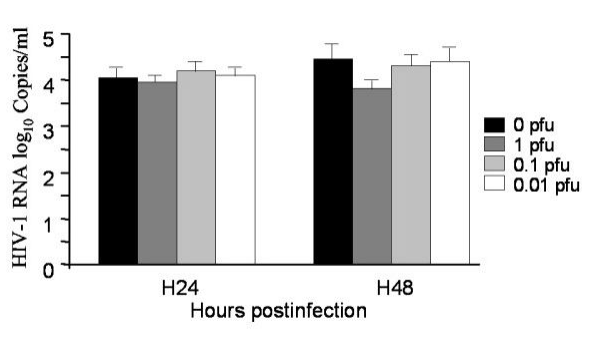
**HIV-1 expression in dually HIV-1 and HSV-2 infected P4P cells**. HIV-1 production in HIV-1 infected P4P cells 24 h and 48 h after HSV-2 superinfection at the MOI of 1 (dark grey), 0.1 (light grey) and 0.01 (white). Values of HIV-1 RNA loads, expressed in log_10 _copies/ml, are the mean of three independent experiments. Control HIV-1 infected HT29 cells not superinfected by HSV-2 are represented in black.

P4P cells, derived from the endometrial epithelium, were also infected in the same conditions as those used for HT29 cells. The rate of HIV-1 infection of P4P cells was around 50%, as determined by the ratio of HIV-1 DNA and albumin levels, suggesting that any viral superinfection would probably produce dually infected cells. As shown in Figure 5, superinfection by HSV-2 did not significantly influence HIV-1 production in P4P cells. The culture supernatants collected 24 and 48 h after HSV-2 superinfection were used to infect Vero cells. After 48 h of infection, no HIV-1 proviral DNA was detected in the cell pellet, while HSV-2 production was confirmed by the detection of HSV-2 DNA in the supernatant (not shown). Co-infected P4P cells did not produce HIV-1 particles able to infect Vero cells. To check the infectiousness of HIV particles produced in the supernatant of co-infected P4P cells, we tested 24 and 48 h culture supernatants from dually HIV-1 and HSV-2 infected P4P cells on uninfected P4P cells for HIV-1 replication. As expected, these supernatants induced HIV productive infection at 24 hours (not shown), thus confirming that supernatants recovered after co-infection of P4P cells contained infectious HIV-1 particles.

Similar results were observed for both epithelial cell lines with R5-tropic strains HIV Ba-L and HIV JR-CSF (not shown). Thus co-infection of intestinal or endometrial epithelial cells with HIV-1 and HSV-2 did not lead to the production of HIV-1 virions with the ability to infect cells naturally non-permissive for HIV-1.

### HIV-1 particles produced from P4P cells expressing HSV-2 gB or gD

In order to avoid the necrosis and apoptosis of epithelial cells consequent to HSV-2 infection, we transfected HIV-1 NDK-infected P4P cells with plasmids coding for gB and gD, which are major glycoproteins involved in fusion of the HSV-2 envelope with the plasma membrane. Culture supernatants obtained 48 h following the transfection were used to infect Vero cells for 24 h. Cell DNA was then extracted, and tested for HIV-1 DNA. Proviral HIV-1 DNA was never detected, suggesting that the expression of gB and gD in HIV-1 infected P4P cells did not modify the phenotype of HIV-1 viral particles such that they could infect Vero cells.

### Viral capture assay

**Figure 6:**
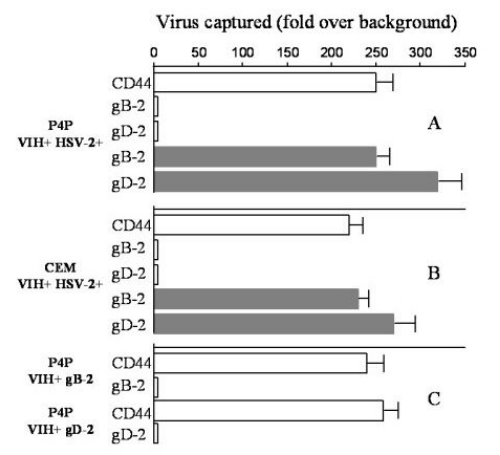
**Viral capture assay**. HIV-1 and HSV-2 capture by antibodies targeted against cell surface CD44 antigen or HSV-2 gB or gD in HIV-1- and HSV-2 dually infected P4P cells (A), CEM cells (B) and HIV-1-infected P4P cells transfected by gB or gD (C). Levels of captured virus were determined by HIV-1 RNA (open square) and HSV-2 DNA (bold square) quantification by real time PCR and compared to capture by a negative control antibody (biotin labeled anti-mouse) to derive fold-over background values. Data are representative of three independent experiments.

In order to assess weteher the colocalization of HSV-2 and HIV glycoproteins can lead to the incorporation of the former into the HIV-1 envelope, we carried out a viral capture assay. Supernatants of HSV-2 and HIV-1 dually infected P4P and CEM cells and of P4P cells infected by HIV-1 then transfected with plasmids coding for gB or gD were first incubated with mouse antibody to gB or gD and biotin-labeled anti-mouse antibody and second with streptavidin microbeads to capture viral particles bearing gB or gD glycoproteins. The positive control capture of HIV-1 particles was assessed by anti-human CD44. Bound viruses were further tested for the detection of HIV-1 RNA and HSV-2 DNA. Capture assays with gB or gD specific antibodies were able to bind HSV-2 virions (Figure 6). No HIV-1 RNA was detected, either from dually infected cells or from gB or gD transfected HIV-1 positive cells, suggesting that HIV-1 particles did not acquire gB or gD glycoproteins during budding (Figure 6).

## Discussion

Sexual transmission of HIV-1 is suspected to be increased when HSV-2 is shed in the genital tract of dually HIV-1- and HSV-2-infected individuals [19,20]. HSV-2 may increase the infectiousness of HIV-infected subjects, by increasing genital HIV load during an HSV-2 genital recurrence [5-8] through the transactivation of HIV-1 LTR by interaction with HSV proteins (ICPO, ICP4) or the production of pro-inflammatory chemokines known to enhance HIV-1 replication [9,10]. The recruitment of activated CD4+ cells [12]that markedly upregulate HIV replication in HSV-infected lesions [13] may also account for the high titer of HIV in genital HSV lesions. Other mechanisms increasing the infectiousness of HIV by HSV-2 may be hypothesized. HIV-1 has been reported to undergo pseudotype formation in vitro when cells are coinfected with HIV-1 and other enveloped viruses by acquiring in the HIV-1 envelope proteins of the co-infecting virus, including human T lymphotropic virus type 1 [21,22], HIV-2 [23], endogenous murine retroviruses [24-26], vesicular stomatitis virus [15], and HSV-1 [14,15]. Such pseudotype formation results in altered cell tropism for the next round of infection [21,22]. Pseudotype formation between HIV-1 and HSV-2 could have an impact on sexual transmission by facilitating HIV-1 epithelial cell infection within the genital mucosa.

In the present study, we investigated whether HIV-1 and HSV-2 can interact at the cellular level by forming HIV-1 hybrid virions pseudotyped with HSV-2 envelope glycoproteins, as was previously reported for HSV-1 [14,15]. We report that HIV-1 produced from HIV-1 and HSV-2 co-infected mononuclear cells and epithelial cells releasing high titers of both HIV-1 and HSV-2 does not acquire an increased tropism to naturally non-permissive cells. We also find no evidence of incorporation into the HIV-1 envelope of gB or gD, which are essential for the binding and entry of HSV-2 [27,28]. In order to avoid the deleterious cytopathic effects of HSV-2 infection, we used plasmids coding for gB and gD. Despite microscopic observations of transfected cells expressing both gp160 and gB or gD and a co-localization of HIV-1 envelope proteins and HSV-2 glycoproteins, HIV-1 virions did not acquire either gB or gD. We cannot completely exclude the formation of HIV-1 pseudotypes by the incorporation of other HSV-2 proteins in the HIV-1 viral membrane. However, our observations demonstrate that no significant level of surface viral proteins involved in HSV-2 cell entry was incorporated, since no HSV-2 tropism was detected in HIV-1 particles. Nor can we exclude pseudotype formation by the integration of HIV-1 genes into the HSV-2 genome or HSV-2 genes into the HIV-1 genome, as was described in chickens with the Marek disease herpesvirus and the reticuloendotheliosis lentivirus, which can both induce T lymphomas in chickens and often coexist in the same animal [29,30]. However such hybrid virions do not acquire a modified cell tropism [29,30]. We observed partial colocalization of gp160 with gB and gD at the plasma membrane level of co-infected cells and HIV-1-infected P4P cells transfected with a plasmid coding for gB or gD. Such slight co-localization may be explained by the major intracellular expression of gB in early endosomes [31] and that the final HSV-2 envelope is thought to be acquired in the trans-Golgi network [28,32] while gp120 is preferentially expressed at the cell surface [33,34]. In addition, the long cytoplasmic domain of gB with 112 amino acids could be sterically inhibitory to be incorporated in a retroviral particle [35-38]. These features are consistent with the lack of gB and gD incorporation into the HIV-1 envelope.

Other studies analyzing interactions between HIV and herpesviruses did not provide evidence for the formation of HIV-1 pseudotypes [40]. Similarly, Van Kuyk and colleagues have reported that HIV-1 obtained from EBV productively infected lymphobastoid cell lines did not acquire the EBV tropism [41]. Toth and colleagues were unable to detect HIV-1/HCMV pseudotypes in supernatant fluids from dually infected syncytiotrophoblasts [40].

Our observations contrast with previous reports on HIV-1/HSV-1 pseudotype formation [14,15]. Thus, cell-free culture supernatant from dually infected lymphocyte cell lines was able to induce HIV productive infection in cells normally resistant to HIV infection, but there was no direct evidence for incorporation of HSV-1 proteins into the HIV-1 envelope [14,15]. Zhu et al reported the co-infection of HIV-producing H9 cells with HSV-1 tsJ12, a temperature sensitive mutant of HSV-1 [15]. Inoculation of HeLa cells with supernatant from the coinfected H9 cells resulted in HIV production, which was not seen with supernatant from H9 cells infected only with HIV-1. However, the level of p24 antigen from H9 cells, which could be higher in co-infected H9 cells as a consequence of HIV transactivation by HSV, was not determined before inoculation of Hela cells. Calistri et al have used HIV-1IIIB produced in H9 cells and HSV-1 to co-infect CEM cells and CD4- Vero cells with the aim of evaluating pseudotype formation [14]. High production of HIV was observed when Vero cells were infected with the CEM_HSV/HIV_cell-free supernatant. However, the levels of HIV replication in CEM_HSV/HIV _and in control CEM_HIV _cell-free supernatants before Vero infection, and those in Vero supernatant infected with the CEM_HIV _cell-free supernatants were not mentioned. In addition, HIV-1 proviral DNA was detected in Vero cells infected by the CEM_HSV/HIV_cell-free supernatant, but the negative control consisted of Vero cells infected with supernatant of H9 cells chronically infected by HIV-1, instead of Vero cells infected with CEM_HIV _cell-free supernatants. Prior HSV-1 infection of CEM cells has been shown to result in a delay of HIV-1 production, due to cell growth inhibition and apoptosis [17]. Thus we cannot exclude a significant release of HIV-1 proviral DNA in CEM_HSV/HIV_supernantant, leading to the false positive detection of HIV-1 proviral DNA in Vero cells.

In conclusion, we find no evidence of in vitro HIV-1/HSV-2 pseudotype formation in mononuclear cells or in epithelial cells. These findings suggest that the existence of HIV-1/HSV-2 pseudotype formation in the genital tract of HIV-1- and HSV-2- co-infected individuals is unlikely, and that genital HIV-1 particles highly produced under the influence of HSV-2 genital replication, as observed in HIV-1- and HSV-2- coinfected women, do not acquire properties of an epithelial cell-tropic virus, such as HSV. Finally, the synergistic interactions between HIV-1 and HSV-2 at the genital level more likely results in increased genital inoculum of both viruses, rather than in phenotype changes of genital HIV-1.

## Materials and methods

### Cells, viruses, and antibodies

P4P cells were kindly provided by Florence Damond. P4P cells are adherent HeLa cells transfected by plasmids coding for CD4 and CCR5 (Barin et al, 2004) and allow infection by both X4 and R5 viruses. P4P cells were grown in Dulbecco modified Eagle medium (DMEM) supplemented with 10% fetal bovine serum (FBS), 500 mg/mL G418, and 100 mg/mL hygromycin B and antibiotics (Penicillin 100 U/ml and streptomycin 100 μg/ml (PS)). Vero cells (African green monkey kidney cells) were grown in DMEM supplemented with 10% FBS and PS. CEM-SS cells were grown in RPMI-1640 medium supplemented with 10% FBS and PS.

HSV-2 strain VR734 was obtained from the American Type Culture Collection (ATCC) (Manassas, VA), HSV-1 MacIntyre Strain was purchased from Advanced Biotechnologies Inc (Columbia, MA), and propagation and titer determination were carried out on Vero cells.

Primary R5-tropic HIV Ba-L, HIV JR-CSF, and X4-tropic HIV NDK were a gift from Pr. F. Barre'-Sinoussi (Institut Pasteur, Paris, France). HIV Ba-L and HIV JR-CSF were produced in primary macrophages; HIV NDK was produced on IL-2-activated peripheral blood lymphocytes. Viral titre was measured by HIV-1 p24 antigen quantification.

Mouse anti-gB and anti-gD monoclonal antibodies (Mabs) were obtained from Serotec (Oxford, UK) and Advanced Biotechnologies Incorporated, respectively. Purified human polyclonal anti-gp160 IgG was described previously [39]. FITC-labeled F(ab')2 goat anti-human IgG, FITC-labeled F(ab')2 rabbit anti-human IgG and TRITC-labelled rabbit anti-mouse IgG were purchased from Jackson Immunoresearch (Cambridgeshire, UK) and Molecular Probes (Cergy Pontoise, France), respectively.

### Plasmids

Plasmids pMM245 and pMM346, coding for HSV-2 gB and gD respectively, were described previously [40].

### Infection assay

P4P and HT29 cells in 12-well plates were infected with 5 ng/ml p24 HIV-1BaL, HIV-1JR-CSF, and HIV-1NDK for 3 h at 37°C and CEM cells were infected with 1 ng/105 cells of p24 HIV-1BaL, HIV-1JR-CSF, and HIV-1NDK. The cells were washed, and were then either cultured for 48 h before superinfection with 1, 0.1 or 0.01 plaque forming unit (pfu)/cell of HSV-2 or transfected with the plasmids pMM245 and pMM346 and cultured for 48 additional hours.

Vero cells in 24-well plates were incubated for 3 h at 37°C with culture supernatants of CEM cells, HT29 cells and P4P cells. After extensive washing, Vero cells were cultured for 24 to 48 h. Supernatants were frozen at -80°C until subsequent analyses and cells were then washed, before the nucleic acid extraction of the cell pellet for the detection of proviral DNA.

### Transfection assay

The transfection assay was performed using 1 μg of plasmid with the transfection agent Fugene^® ^(Roche Applied Science, Meylan, France). The protein expression was assessed 48 h following the transfection by intracellular staining as described below.

### Intracellular staining of HSV-2 gB and gD and HIV-1 gp-160

Cells were washed, fixed with paraformaldehyde (4% in phosphate-buffered saline [PBS]) for 10 min at 4°C before permeabilization in PBS with 0.5% Triton X-100 and 10% sucrose at room temperature. After several washes with PBS, cells were incubated for 30 min with human anti-gp160 IgG, or with mouse anti-gB or anti-gD Mabs diluted in PBS with 1% bovine serum albumin. FITC-labeled F(ab')2 rabbit anti-human IgG and TRITC-labelled rabbit anti-mouse IgG were further added at a dilution of 1/50 for 30 min at room temperature. The coverslips were mounted in Mowiol (Sigma, St. Louis, Mo.) and observed by confocal microscopy. Observations were made by sequential acquisition with a Zeiss LSM510 System, mounted on an Axiovert 100 M optical microscope (Carl Zeiss AG, Oberkochen, Germany), using a planapochromat x63, 1.4 numerical aperture oil immersion objective. Optical sections were acquired, each one with an image resolution of 512 × 512 pixels.

### Viral capture assay

To assess the incorporation of HSV-2 envelope glycoproteins into the HIV-1 membrane, we carried out a viral capture assay to bind viruses bearing gB or gD as follows. HIV-1, HSV-2, and mixture of HIV-1 and HSV-2 virions, produced in gB- or gD-expressing P4P cells (200 μl), were incubated first with 1 μg of mouse anti-gB or mouse anti-gD and second with 1 μg of anti-mouse biotinylated antibody (Sigma-Aldrich, Lyon, France) for 30 min at room temperature to bind viruses bearing HSV-2 glycoproteins. Similarly, to bind HIV-1 paricles, supernatants were incubated with mouse anti -human CD44 (BD Biosciences, Le Pont de Claix, France) and with 1 μg of anti-mouse biotinylated antibody (Sigma-Aldrich) as described [41], since CD44 has been demonstrated to be the most effective host cell marker for the capture of HIV-1 from patient samples and culture-derived virus, independent of the origin of the virus [41]. To each sample were added 25 μl μMACS™ Streptavidin MicroBeads (Miltenyi Biotec, Auburn, CA) and the binding reaction was incubated an additional 10 min at room temperature. Antibody-bound viruses were then captured by magnetic separation according to the manufacturer's protocol. Briefly, μMACS columns were placed in the μMACS™ magnetic separator attached to a magnetic MultiStand. Each column was prepared by prewetting with 100 μl protein equilibration buffer and rinsing twice with 100 μl PBS containing 2% fetal bovine serum (PBS/FBS). The entire volume of the virus capture reaction mixture (200 μl) was then applied to the column and allowed to drain completely. The columns were washed six times with 200 μl volumes of PBS/FBS. Fifty microliters of an appropriate lysis buffer (see below) were then added to the column, allowed to stand for 5 min at room temperature, and then followed by an additional 150 μl of the same buffer. The eluates were then processed for nucleic acid purification as follows. Nonspecific viral binding was assessed by adding anti-mouse biotinylated antibody to virus before capture (negative control).

### Nucleic acid purifications

Nucleic acids were extracted with an extraction protocol on silica column system (High Pure Viral Nucleic Acid kit, Roche Diagnostics, Mannheim, Germany) according to the manufacturer's recommendations. Total nucleic acids were eluted in 100 μl of RNAse- and DNAse-free water.

### Detection and quantification of HIV-1 RNA and DNA

HIV-1 RNA and DNA quantification PCR were carried out by real time using the forward NEC152 (5'-GCCTCAATAAAGCTTGCCTTGA-3') and reverse NEC131 (5'-GGCGCCACTGCTAGAGATTTT-3') primers and the exonuclease probe (5'-FAM-AAGTAGTGTGTGCCCGTCTGTTRTKTGACT-TAMRA-3') in the long terminal repeat gene [42] on the Light cycler instrument 1.5 (Roche Diagnostics) as described [43]. For HIV RNA quantification, the positive control consisted of culture supernatant of HIV-1 subtype A strain. For HIV DNA quantification, the positive control consisted of DNA purified from T lymphoblastoid cell line 8E5 (NIH, ATCC 8993), containing a single copy of HIV-1 LAV DNA provirus per cell. The level of albumin DNA copies, quantified by real time PCR as described previously [44], was used as an endogenous reference to normalize the variations in cell number or DNA extraction. HIV-1 RNA was expressed in copies/ml, and HIV-1 DNA proviral load as the number of HIV-1 DNA copies per 10^6 ^cells.

### Detection and quantification of HSV-2 DNA

HSV-2 DNA was detected and quantified by real time PCR, using HSV pol F (5' GCTCGAGTGCGAAAAAACGTTC 3') and HSV pol R (5' TGCGGTTGATAAACGCGCAGT 3') as primers, and HSV-2 FLU (5' GCGCACCAGATCCACGCCCTTGATGAGC-FLUOR) and HSV-2 LCR (5' LC-Red 640-CTTGCCCCCGCAGATGACGCC-phos) as fluorescence labelled probes, as previously described [45]. The positive control consisted of the 324-bp pcDNA3.1/HisC-ul30hsv plasmid containing the targeted nucleotides 2660 to 2963 of the UL30 gene [43]. A standard graph of the Ct values was obtained from serial dilutions (10 to 10^7 ^copies) of the plasmid.

### Quantification of HIV-1 p24 antigen

Levels of HIV-1 p24 antigen released in culture supernatants were determined by a commercial enzyme-linked immunosorbent assay (ELISA) (INNOTEST^® ^HIV Antigen mAb, Innogenetics, Lille, France), with a cutoff value of 1 pg/ml.
